# Bright’s Disease of the Kidney

**Published:** 1860-02

**Authors:** Geo. Johnson

**Affiliations:** Physician to King’s College Hospital


### BRIGHT'S DISEASE OF THE KIDNEY,

### THE FORMS AND STAGES, WITH ESPECIAL REFERENCE TO DIAGNOSIS AND PROGNOSIS.

—

BY DR. GEO. JOHNSON, PHYSICIAN TO KING'S COLLEGE HOSPITAL.

—

[From Ranking's Abstract.]

The author considers that the *oneness* of Bright's disease is inconsistent with the clinical history and the morbid anatomy of the kidney in its various conditions. A large Bright's kidney usually remains large to the end, and a contracted Bright's kidney has not passed through a previous stage of enlargement. The chief points of distinction between the large white Bright's kidney and the contracted kidney are the following:—

1. The urine secreted by the large kidney is less abundant, of higher density, more constantly and copiously albuminous, and contains small waxy casts with or without oil, but not the granular casts which are thrown off from the tubes of the contracting kidneys.

2. The minute anatomy of the two kidneys is different.—In the contracting kidney, the gland cells becomes disintegrated and washed away, leaving the tubes denuded. In the large white kidney, the cells for the most part remain attached to the basement membrane, and undergo changes varying from a slight glanular opacity to a complete oily degeneration, or they may at length become replaced by an albuminous or a fibrinous material which fills and obstructs the tubes.

3. Another important distinction between the two forms of disease consists in the relative frequency of dropsy as a symptom. There are few exceptions to the rule, that patients who die with a large Bright's kidney have had dropsy. In only two out of twenty-six fatal cases in the author's experience, had dropsy been wanting at some period of the patient's history; whereas, of thirty-three fatal cases of contracted kidney, there had been dropsy in only fourteen, and in the most of these cases the dropsy was very slight and partial.

The author remarked that if all the contracted Bright's kidneys have passed through a previous state of enlargement, it is difficult to understand how the majority of those patients who have reached this final stage of renal degeneration can escape the dropsy which, in a greater or less degree, troubled nearly all those who die in what is by some writers assumes to be an earlier stage of the same disease. The rule is, that a large Bright's kidney remains so to the end, and that a contracted kidney has not previously been enlarged. But there are exceptions to this rule. There are cases of Bright's disease in which the kidneys, having become enlarged, subsequently undergo a process of contraction in a greater or less degree. These cases, however, are so exceptional in many of their most important features, that they afford a remarkable confirmation of the doctrine, that in ordinary cases the contracted Bright's kidney is not an advanced stage of a previously enlarged kidney, but the result of a distinct series of morbid changes. The cases in which contraction of the kidney has followed upon enlargement may be divided into three classes: First, there are cases in which the weight and size of the kidney are found to be increased after death; yet in the cortical portion of the gland there are unquestionable indications of atrophy and contraction. The author said he had met with six cases of this kind, and showed a drawing of the kidney from one case. The gland was large, pale, and waxy, but the cortical portion decidedly wasted. In the second class of cases, the contraction of a pale, waxy kidney had proceeded farther, so that the size and weight were reduced below the average of the healthy gland. The third class of cases are those in which a kidney, having become enlarged and *fatty*, has subsequently contracted; the fat granulations being still visible in the atrophied kidney. During a period of fourteen years the author has met with five examples of contracted fat kidney. In two of these cases the period of transition from a large fat kidney to a contracting kidney was clearly indicated by the microscopic characters of the urine. The large waxy and granular casts were observed to take the place of the small waxy and oily casts which had been present during the earlier periods of the disease. One of these cases was under observation for a period of ten years. The chief points of distinction between a kidney which has been wasted by the chronic desquamative disease and one which has contracted after waxy enlargement were referred to, and illustrated by drawings of two forms of disease.

---

LONGEVITY.—The following may be seen on a gravestone in Derwin (Denbigshire) church-yard: "Husband died, aged 103; wife died, aged 98; their son died, aged 97; their daughter, aged 107; their grandson, aged 98; total, 499; average 99½.

